# Knowledge, attitudes, and practices toward depression among physicians in Sudan

**DOI:** 10.1002/brb3.3321

**Published:** 2023-11-05

**Authors:** Sohaib Mohammed Mokhtar Ahmed, Elnazier Mohammed Ibrahim Mohamed Zain, Osama Saeed Osman

**Affiliations:** ^1^ Faculty of Medicine and Health Science Gadarif University Gadarif Sudan; ^2^ Gadarif Regional Institute of Endemics Diseases (GRIED) Gadarif University Gadarif Sudan

**Keywords:** depression, knowledge, physicians, Sudan

## Abstract

**Objectives:**

The present study assessed the knowledge, attitudes, and practices of physicians in Sudan regarding depression.

**Methods:**

A cross‐sectional study was conducted among physicians who practiced at public and private health facilities in Sudan in December of 2022. An online Google form questionnaire was administered that included knowledge, practice, and sociodemographic sections of the depression attitude questionnaire. The link to the questionnaire was sent to a convenience sample of physicians through a variety of methods including social media.

**Results:**

A total of 407 physicians completed the questionnaire. The majority (81.1%) had no formal training on mental health after graduation. A total of 43.0% reported difficulties in differentiating between unhappiness and clinical depression, although 48.6% indicated that they could differentiate between chemical and psychological causes for depression. Half (50.4%) did not feel comfortable dealing with depressed patients. Nearly 70% indicated that psychotherapy was a better option for treating these patients than antidepressants, but only 45.7% had any mental health services at their health facility. Physicians with prior mental health training (both pre‐ and postgraduate training) were more likely to provide treatment options for depressed patients.

**Conclusions:**

This study indicates a moderate knowledge among physicians about depression, but also significant concern regarding poor attitudes and practices held toward the treatment of depression, and a lack of training. These findings highlight the urgent need for the training of physicians in the diagnosis and treatment of depression in Sudan.

## Abstract 2

Graphical Abstract

## 1 INTRODUCTION

Depression is a debilitating illness that significantly affects an individual's energy and behavior. It is characterized by persistently low mood and diminished interest in activities, and it is also associated with an increased risk of chronic diseases (Cohen et al., 2015). Major depressive disorder is the leading cause of disability in the United States (World Health Organization, 2001). Furthermore, depression is one of the most prevalent mental health conditions globally. According to the World Health Organization, approximately 322 million people were affected by depression in 2015, with women being more susceptible than men (World Health Organization, 2017). Nearly one in eight individuals has experienced depression at least once in their lifetime, and each year, around 18.8 million adolescents suffer from a depressive illness (Okasha, 2002).

Unfortunately, mental health conditions receive insufficient attention from most governments, resulting in underdeveloped mental health services (Chen et al., 2011; Pence et al., 2012). The primary care setting is where most individuals with depression seek treatment (Ornstein et al., 2000; Simon, 1998). Despite the availability of effective treatments in this setting, less than half of individuals with depression receive a proper diagnosis and even fewer receive adequate treatment (Pincus et al., 2001). Insufficient care has been attributed to the barriers faced by physicians when treating patients with depression (Simon, 2003). Given the social and economic burden of depression, enhancing the quality of depression care in primary care settings becomes an imperative goal (World Health Organization, 2010).

The medical education system in Sudan is unique, with its challenges and strengths. Historically, the curriculum has been heavily influenced by the British system but has evolved over the years to cater to the nation's specific needs. Although the curriculum covers a broad range of medical topics, mental health has often been given less emphasis. This might be attributed to cultural perceptions, lack of resources, or other systemic factors. As a result, many physicians graduate with limited exposure to psychiatric conditions, potentially leading to gaps in knowledge and possibly fostering negative or uninformed attitudes toward mental illnesses.

Depression is now widely recognized as a significant public health issue worldwide, and the importance of primary care‐based support for individuals with depression is well‐established. The World Health Organization has reported that depression is the primary cause of disability and the fourth‐largest contributor to the global burden of disease. It also ranks as the second leading cause of disability‐adjusted life years among individuals aged 15–44 for both sexes. However, a substantial number of people with depression remain undiagnosed or do not receive adequate support or treatment (World Health Organization, 2010).

Physicians play a critical role in the healthcare system as primary care providers and decision‐makers in the diagnosis, treatment, and management of various health conditions, including depression. Particularly in primary care settings, physicians are often the initial point of contact for individuals seeking mental health support. They hold crucial responsibilities in recognizing signs and symptoms of depression, providing appropriate interventions or referrals, and promoting mental health awareness. Understanding the knowledge, attitudes, and practices of physicians can shed light on potential barriers, challenges, and opportunities for improving depression care in Sudan. Therefore, this study focuses on examining the knowledge, attitudes, and practices of physicians in health facilities in Sudan regarding depression. We specifically concentrate on depression because it is the most prevalent mental health disorder worldwide and its prevalence is increasing in Sudan and globally. The urgency for this study is heightened by the current economic, military, and political conflicts in Sudan, which may have a negative impact on people's mental well‐being. Enhancing mental health services can have a significant impact on the overall management of depression, tailored to our specific context, and potentially improve mental health care in Sudan.

## 2 METHODS

### 2.1 Study design

We conducted a cross‐sectional study involving all physicians working in public and private health facilities in Sudan. The study was conducted from December 9 to 30, 2022.

### 2.2 Study participants and setting

This study took place in Sudan, located in northeast Africa, covering the south, north, east, west, and central regions of the country. All physicians working in public or private health facilities in Sudan, aged at least 21 years, and willing to participate were included in the study. Psychiatrists, anesthesia technicians, nurses, pharmacists, and undergraduate medical students were excluded.

### 2.3 Sample size and sampling technique

Due to the lack of an official number of doctors in Sudan, we employed the unknown population sample size formula with a population proportion of 50%, a margin of error of 5, and a confidence interval of 95%. The minimum sample size required was calculated to be 385 participants, and we ultimately obtained a sample of 407 participants.

Convenience sampling was utilized as the official record of physician numbers and information was not feasible. We employed an online Google Form questionnaire distributed through medical students serving as data collectors across different states of the country. Additionally, the questionnaire was shared with physicians through social media groups such as WhatsApp, Telegram, and Facebook.

### 2.4 Study instruments

The structured and self‐administered questionnaire consisted of four sections. The first section collected sociodemographic information, including sex, specialty, and experience. The second section comprised 10 questions assessing knowledge about the Diagnostic and Statistical Manual of Mental Disorders, 5th Edition (DSM‐V), and pharmacological treatment of depression. These questions were derived from previous studies (Alanazi et al., 2022; Mulango et al., 2018).

The third section utilized the depression attitude questionnaire (DAQ) to evaluate the attitudes of physicians toward depression. The DAQ was originally developed for use in the United Kingdom and has been successfully employed by various health professionals in other countries (Haddad et al., 2016; Mbatia et al., 2009; Mulango et al., 2018). The original DAQ consisted of 20 self‐reported items measured on a visual analogue scale ranging from “strongly agree” to “disagree” (Haddad et al., 2007).

The fourth section assessed the practices, training, and availability of psychiatric services. The questionnaire's internal consistency reliability, based on a sample of 407 participants, was assessed using Cronbach's alpha test, yielding a value of .78, indicating good reliability.

### 2.5 Data management

To ensure data integrity, we employed robust data collection methods with form logic and validation checks. Access to the data was restricted to authorized personnel, and transparent statistical analyses adhering to established guidelines were performed. Data analysis was conducted using Jamovi version 2.3.21.0 (The Jamovi Project, 2023). Categorical data were described using frequencies and percentages, whereas numerical data were presented as medians and interquartile ranges. Sociodemographic factors were compared with randomly selected questions using the chi‐square test or Fisher's exact test, as appropriate. A *p*‐value of .05 or less was considered statistically significant.

## 3 RESULTS

### 3.1 Sample characteristics

The study included 407 physicians with a median age of 27 years old (IQR = 5). Of these, 178 (43.7%) were male. The majority, 330 (81.1%), did not receive any formal mental health training after graduation, whereas 221 (54.3%) did not receive such training during their undergraduate period. About 178 (43.7%) had less than 2 years of experience, 130 (31.9%) had 2–6 years of experience, and 99 (24.3%) had more than 6 years. The majority of participants were House Officers and general practitioners (GPs), accounting for 41.5% and 33.7%, respectively. Sociodemographic characteristics are displayed in Table 1.

**TABLE 1:** Sociodemographic characteristics of the participants.

Variables	Categories	*N*	%
Sex	Male	178	43.7
	Female	229	56.3
Specialty	Family physicians	6	1.5
	Physicians	28	6.9
	Surgeons	20	4.9
	GPs	137	33.7
	Dermatologists	4	1
	Pathologists	7	1.7
	House officers	169	41.5
	Obstetricians	14	3.4
	Pediatricians	17	4.2
	Others	5	1.2
Experiences	Less than 2 years	178	43.7
	From 2 to 6 years	130	31.9
	More than 6 years	99	24.3
Residence	East Sudan	130	31.9
	West Sudan	39	9.6
	Middle Sudan	210	51.6
	North Sudan	14	3.4
	South Sudan	14	3.4

Abbreviation: GP, general practitioner.

### 3.2 Knowledge about DSM‐V criteria

The majority of physicians selected the correct items in the DSM‐VI questions. For example, 86% agreed with the statement “Depression affects the ability of patients to think or concentrate,” and 86.7% agreed with the statement “Depressed patients show a decrease in interest or pleasure in all activities in their day, nearly every day.” Table 2 shows the responses for DSM‐V related questions.

**TABLE 2:** Responses to Diagnostic and statistical manual of mental disorders (5th ed.) (DSM‐V) criteria items.

Statements	Agree, *N* (%)	Disagree, *N* (%)	Do not know, *N* (%)
Depressed patients feel worthless and inappropriate guilt nearly every day	290 (71.3)	51 (12.5)	66 (16.2)
Depression affects the ability of patients to think or concentrate	350 (86)	39 (9.6)	18 (4.4)
Depression can lead to suicide or suicide attempts	380 (93.4)	14 (3.4)	13 (3.2)
Patients with depression show significant weight loss or gain and significant decrease or increase in appetite	363 (89.2)	26 (6.4)	18 (4.4)
Depressed patients feel tired and have no energy to do anything, nearly every day	348 (85.5)	37 (9.1)	22 (5.4)
Depressed patients show decrease in interest or pleasure in all activities in their day, nearly every day	353 (86.7)	32 (7.9)	22 (5.4)

### 3.3 Beliefs about causes of depression

More than half (55.8%) of physicians disagreed that depression affects people of specific ages. Slightly more than a third (37.3%) agreed that the majority of depression cases they encounter are the result of recent misfortune. In addition, 55.3% selected biochemical abnormalities as the cause of more severe depression, and 60.4% stated that depression is a natural part of getting older. However, 51.4% agreed that poor stamina is a cause of depression. Table 3 details the responses from the DAQ.

**TABLE 3:** Responses to depression attitudes questionnaire (DAQ) items.

Statements	Agree*N* (%)	Disagree *N* (%)	Do not know *N* (%)
During the last 5 years, I have seen an increase in the number of patients presenting with depressive symptoms	292 (71.7)	40 (9.8)	75 (18.4)
The majority of depression cases I see originated from recent misfortune	152 (37.3)	64 (15.7)	191 (46.9)
Most depressive disorders improve without medication	176 (43.2)	174 (42.8)	57 (14.0)
Biochemical abnormality is at the basis of more severe depression	225 (55.3)	49 (12.0)	133 (32.7)
It is difficult to differentiate unhappiness from a clinical depressive disorder that needs treatment	175 (43.0)	179 (44.0)	53 (13.0)
It is possible to distinguish two groups of depression, one psychological in origin and the other caused by biochemical mechanisms	198 (48.6)	75 (18.4)	134 (32.9)
Becoming depressed is a way that people with poor stamina deal with life difficulties	209 (51.4)	102 (25.1)	96 (23.6)
Depressed patients are more likely to have experienced deprivation in early life than other people	256 (62.9)	52 (12.8)	99 (24.3)
I feel comfortable dealing with depressed patients	131 (32.2)	205 (50.4)	71 (17.4)
Depression reflects a characteristic response which is not amendable to change	105 (25.8)	175 (43.0)	127 (31.2)
Becoming depressed is a natural part of becoming old	109 (26.8)	246 (60.4)	52 (12.8)
The primary health‐care worker could be a useful person to support depressed patients	321 (78.9)	60 (14.7)	26 (6.4)
Working with depressed patients is heavy going, tedious	235 (57.7)	83 (20.4)	89 (21.9)
There is little to be offered to depressed patients who do not respond to what primary health‐care workers do	147 (36.1)	138 (33.9)	122 (30.0)
It is rewarding to spend time looking after depressed patients	235 (57.7)	74 (18.2)	98 (24.1)
If depressed patients need antidepressants, they are better off with psychiatrists than with primary health‐care workers	330 (81.1)	39 (9.6)	38 (9.3)
Antidepressants usually produce a satisfactory result in the treatment of depressed patients in general practice	249 (61.2)	75 (18.4)	83 (20.4)
Psychotherapy for depressed patients should be left to a specialist	328 (80.6)	49 (12.0)	30 (7.4)
If psychotherapy were freely available, this would be more beneficial than antidepressants for most depressed patients	281 (69.0)	69 (17.0)	57 (14.0)
Patients with depression are discriminated in by the general public and avoided	224 (55.0)	109 (26.8)	74 (18.2)

### 3.4 Attitudes toward depression identification and prognosis

More than a third (43.2%) of physicians believed that depressed patients can improve without medication. 43% of participants found it difficult to differentiate between unhappiness and clinical depression. However, nearly half of them (48.6%) could differentiate between psychological and biochemical causes of depression.

### 3.5 Knowledge and attitudes toward depression treatment

More than half (55.3%) agreed that amitriptyline is an antidepressant, whereas 38.6% did not know about it. Furthermore, two thirds (66.1%) opposed methotrexate as an antidepressant. However, one third (35.6%) agreed that carbamazepine is a first‐line antidepressant, whereas other third (32.4%) did not know it. Overall, 44.5% agreed with the possibility of a physician prescribing a selective serotonin reuptake inhibitor to a newly diagnosed patient.

Half of the physicians (50.4%) did not feel comfortable dealing with depressed patients, although more than half (57.3%) declared that it is rewarding to spend time looking for depressed patients. If an antidepressant is needed, the majority (81.1%) preferred a psychiatrist over a primary health‐care worker, and the same majority (80.6%) preferred a specialist over a primary health‐care worker for psychotherapy. Although more than two thirds (69%) agreed that psychotherapy is more effective than antidepressants for most depressed patients, 55% of the participants mentioned the stigma of depression. More details are shown in Table 3.

### 3.6 Practices and availability of psychiatric services

The majority (71.7%) reported an increasing number of patients presenting with depression symptoms. More than half (57.2%) agreed to provide management options if they identified any person with depression. Only 45.7% had mental health services available in their health facility, whereas the majority (91.9%) recognized the need for doctors to receive mental health training to improve the care of depressed patients. The majority (81%) chose to refer patients with depressive disorder to a psychiatrist. Overall, 20% of physicians stated that they had not dealt with any depressed patients before, whereas 40% stated that they had dealt with them occasionally, and only 5% reported always having contact with depressed patients. Table 4 shows the frequencies of prescriptions and the availability of psychotropic drugs.

**TABLE 4:** Prescription and availability of psychotropic drugs.

Statements	Always, *N* (%)	Sometimes, *N* (%)	Never, *N* (%)	I do not know, *N* (%)
Have you ever prescribe a psychotropic drug for depression?	13 (3.2)	69 (17)	290 (71.3)	35 (8.6)
Psychotropic drugs are available at pharmacies with your health area?	62 (15.2)	200 (49.1)	47 (11.5)	98 (24.1)

### 3.7 Associations with demographic characteristics

Those who received prior training during their undergraduate studies were significantly more likely to provide management options if they identified a patient with depression (*χ* ^2^ = 10.828, *p* = .003). However, no significant differences were found in the preference for psychotherapy over antidepressants (*χ* ^2^ = .062, *p* = .4), the belief that poor stamina is a cause of depression (*χ* ^2^ = .016, *p* = .9), or the ability to distinguish unhappiness from clinical depression (*χ* ^2^ = .016, *p* = .9).

Physicians with prior training after graduation were more likely to know that carbamazepine is an antidepressant (*χ* ^2^ = 4.144, *p* = .021). They were also more likely to provide management options for depressed patients (*χ* ^2^ = 6.635, *p* = .012), but no significant differences were found in the remaining items.

Physicians with less than 2 years of experience had more difficulties differentiating between unhappiness and clinical depression, whereas those with more than 6 years had fewer difficulties (*χ* ^2^ = 7.879, *p* = .007). Furthermore, those with more than 6 years of experience were more likely to provide management options for depressed patients (*χ* ^2^ = 4.222, *p* = .037). However, experience did not result in significant differences for the remaining items.

## 4 DISCUSSION

This study assessed the knowledge, attitudes, and practices of physicians regarding depression in Sudan. The findings reveal that the majority of physicians demonstrated good knowledge about depression symptoms based on their responses to the DSM‐V criteria, a recent standardized tool used in depression diagnosis (O'Connor et al., 2016). However, some misconceptions about the causes of depression, such as misfortune and poor stamina, were identified. The study also highlights slightly positive attitudes among the participants, despite their minimal training and poor practice.

In this study, physicians in Sudan exhibited misconceptions regarding the causes of depression. Approximately 55% of them believed that people with poor stamina are more prone to depression. This misconception was also observed among physicians in Cameroon (Mulango et al., 2018) and Taiwan (Liu et al., 2008), as well as among GPs in Pakistan and Nigeria (Haddad et al., 2016; James et al., 2012). Insufficient psychiatric training in these countries may contribute to such notions, as the majority of our physicians did not receive any training after graduation, which is consistent with physicians in Cameroon and Pakistan.

The study also found that physicians had a good understanding of the DSM‐V criteria for depression, these criteria are essential for improving patients' treatment and controlling the use of antidepressants (O'Connor et al., 2016), and the majority correctly identified key symptoms, such as the impact of depression on patients' ability to think or concentrate and the decrease in interest or pleasure in activities. This knowledge is essential for accurate diagnosis and appropriate treatment planning. The participants also expressed agreement with the effectiveness of pharmacological and psychotherapy treatments for depression, with two thirds considering psychotherapy as more effective than antidepressants, similar to physicians in Cameroon and India (Mulango et al., 2018; Almanzar et al., 2014).

The majority of physicians reported an increase in the number of patients exhibiting depressive symptoms over the last 5 years, indicating a rapid rise in depression among the Sudanese population. This trend aligns with recent studies in Sudan that have identified an increased prevalence of depression among specific population categories, such as patients with type 2 diabetes mellitus (Omar et al., 2021) and end‐stage renal disease (Elkheir et al., 2020). To better recognize and treat depressed patients, it is crucial for physicians to have high confidence. However, a substantial proportion of physicians reported feeling uncomfortable dealing with depressed patients, consistent with previous studies conducted in other countries (Cruz et al., 2019; Mbatia et al., 2009), which may reflect the complexity and challenges associated with managing mental health conditions.

In terms of knowledge and attitudes toward depression treatment, the findings suggest room for improvement. Although a majority recognized amitriptyline as an antidepressant, there was misconception with carbamazepine, which is a mood stabilizer that may have potential use in depressive phases of bipolar disorder or its adjunctive role in refractory depression, but it is not a first‐line antidepressant (Maan et al., 2023). This indicates a need for ongoing education about the range of available antidepressant options. Previous studies have also shown that more than half of depressed patients continue to experience residual symptoms after being prescribed antidepressants by a psychiatrist, highlighting the need to improve knowledge when selecting antidepressant medications (Pitanupong & Sataporn, 2022).

In addition to the limited practices of physicians regarding psychiatric services, as the majority did not prescribe any psychotropic drugs for depression, our study highlighted the deficiencies in psychiatric services in Sudan. Less than half of the participants confirmed the availability of psychiatric services in their health facilities, and even fewer had access to psychotropic drugs in their facility pharmacies. These facts indicate a serious problem considering the increasing prevalence of depression. A study in eastern Sudan demonstrated an increase in depression prevalence among patients with type 2 diabetes mellitus (Omar et al., 2021), as well as among patients with end‐stage renal disease (Elkheir et al., 2020). Improving psychiatric services is crucial to mitigate the impact of this problem, as depression can exacerbate the severity of chronic diseases and hinder patients' recovery (Cohen et al., 2015).

Stigma against depression can deter patients from seeking professional help, negatively impacting psychiatric services (Barney et al., 2006). More than half of the physicians in our study held stigmatizing attitudes toward depression, similar to previous studies conducted in India (Almanzar et al., 2014; Mahla & Gandhi, 2022) and among nurses in the Philippines (Cruz et al., 2019). Effective training on mental disorders is necessary to address this problem.

This study demonstrated the lack of training among physicians in Sudan. Training nonspecialists in the field of mental health is an effective way to expand the global capacity for mental health care. This strategy can improve the knowledge, attitude, skills, and confidence of health workers, as well as the clinical practice and patient outcomes (Caulfield et al., 2019), so it is important to improve the training services in Sudan in order to improve mental health care.

## 5 LIMITATIONS

This study had several limitations. First, a nonrandom sampling method (convenience sampling) was used, which may introduce bias. Additionally, the response rate could not be calculated accurately due to the distribution of the questionnaire through social media. The geographic distribution was limited, with only 14 participants from the north and an equal number from the south of Sudan. Moreover, the majority of our participants were GPs and house officers, which limits the representation of various specialties. On a positive note, we utilized the DAQ for depression attitudes, a tool previously used in many studies in other countries (Botega & Silveira, 1996; Mahla & Gandhi, 2022; Mbatia et al., 2009), allowing for comparisons with findings from other nations.

## 6 CONCLUSION

This study reveals a moderate degree of knowledge among physicians regarding depression. However, significant concerns arise from the poor attitudes and practices observed in their treatment of depression, coupled with a lack of training. Given the increasing number of depressed patients and the current complex situations in Sudan that may worsen the mental health status of the population, we recommend the establishment and implementation of a national mental health program and an awareness education program on mental health illnesses to improve overall mental health in Sudan.

## AUTHOR CONTRIBUTIONS

Sohaib Mohammed Mokhtar Ahmed contributed to the conception and design of the study, acquisition of data, data analysis and interpretation, and drafting and critically revised the manuscript. Elnazier Mohammed Ibrahim Mohamed Zain contributed to the conception and design of the study, acquisition of data, and drafting the manuscript. Osama Saeed Osman contributed to the design of the study, drafting, and critically revised the manuscript.

## CONFLICT OF INTEREST STATEMENT

The authors declare that they have no conflicts of interest regarding this research study or its publication.

### PEER REVIEW

The peer review history for this article is available at https://publons.com/publon/10.1002/brb3.3321.

## Data Availability

The data used in this study are not publicly available due to privacy concerns. Interested parties can request access by contacting the corresponding author, subject to applicable regulations and data protection policies.
